# Chemical Composition, Antioxidant and Anti-Inflammatory Activities of Clary Sage and Coriander Essential Oils Produced on Polluted and Amended Soils-Phytomanagement Approach

**DOI:** 10.3390/molecules26175321

**Published:** 2021-09-01

**Authors:** Robin Raveau, Joël Fontaine, Anthony Verdin, Loris Mistrulli, Frédéric Laruelle, Sophie Fourmentin, Anissa Lounès-Hadj Sahraoui

**Affiliations:** 1 Unité de Chimie Environnementale et Interactions sur le Vivant (UCEIV), Université du Littoral Côte d’Opale, UR 4492, SFR Condorcet FR CNRS 3417, 50 rue Ferdinand Buisson, 62228 Calais, France; robin.raveau@univ-littoral.fr (R.R.); joel.fontaine@univ-littoral.fr (J.F.); frederic.laruelle@univ-littoral.fr (F.L.); 2 Unité de Chimie Environnementale et Interactions sur le Vivant (UCEIV), Université du Littoral Côte d’Opale, UR 4492, SFR Condorcet FR CNRS 3417, 59140 Dunkerque, France; anthony.verdin@univ-littoral.fr (A.V.); Loris.Mistrulli.Etu@univ-lemans.fr (L.M.); sophie.lamotte@univ-littoral.fr (S.F.)

**Keywords:** essential oils, aromatic plants, antioxidant, anti-inflammatory, polluted soils, phytomanagement

## Abstract

The potential of essential oils (EO), distilled from two aromatic plants—clary sage (*Salvia sclarea* L.) and coriander (*Coriandrum sativum* L.)—in view of applications as natural therapeutic agents was evaluated *in vitro*. These two were cultivated on a trace element (TE)-polluted soil, as part of a phytomanagement approach, with the addition of a mycorrhizal inoculant, evaluated for its contribution regarding plant establishment, growth, and biomass production. The evaluation of EO as an antioxidant and anti-inflammatory, with considerations regarding the potential influence of the TE-pollution and of the mycorrhizal inoculation on the EO chemical compositions, were the key focuses. Besides, to overcome EO bioavailability and target accession issues, the encapsulation of EO in *β*-cyclodextrin (*β*-CD) was also assessed. Firstly, clary sage EO was characterized by high proportions of linalyl acetate (51–63%) and linalool (10–17%), coriander seeds EO by a high proportion of linalool (75–83%) and lesser relative amounts of *γ*-terpinene (6–9%) and *α*-pinene (3–5%) and coriander aerial parts EO by 2-decenal (38–51%) and linalool (22–39%). EO chemical compositions were unaffected by both soil pollution and mycorrhizal inoculation. Of the three tested EO, the one from aerial parts of coriander displayed the most significant biological effects, especially regarding anti-inflammatory potential. Furthermore, all tested EO exerted promising antioxidant effects (IC_50_ values ranging from 9 to 38 g L^−^^1^). However, EO encapsulation in *β*-CD did not show a significant improvement of EO biological properties in these experimental conditions. These findings suggest that marginal lands polluted by TE could be used for the production of EO displaying faithful chemical compositions and valuable biological activities, with a non-food perspective.

## 1. Introduction

Soil pollution by trace elements (TE) is attracting global concern due to their toxicity, persistency, and ability to negatively affect soil quality, plant growth, and food quality, as well as animal and human health. For these reasons, the reclamation of TE-polluted soils should be considered as a primary objective worldwide [1,2]. In that respect, phytotechnologies have emerged during recent decades as promising tools to remediate TE-polluted areas. Based on the use of plants and their associated microorganisms, phytotechnologies are considered as environmental-friendly, cost-effective, and benefit from a high public acceptance [1,3,4]. One of the main strategies, referred to as phytostabilisation, resides in the immobilisation of the pollutants in the plant rhizosphere to reduce their bioavailability and alleviate wind and water erosion phenomena [5,6]. However, increased concentrations of TE may impair plant growth and weaken the success of the method [1,6]. Phytostabilisation can hence be supported through the addition of amendments, aiming at further reducing TE bioavailability, while improving soil quality and plant’s growth [7]. Among the available amendments, microbial inoculants and in particular arbuscular mycorrhizal fungi (AMF), able to develop symbiotic associations with most of the terrestrial plants, have long been described for their contribution towards an improved plants’ fitness in agroecosystems [8,9]. Furthermore, their contribution to TE immobilization in the rhizosphere may represent a valuable addition to the technology [10,11]. However, one of the major obstacles that aided phytostabilisation is facing resides in the lack of economic profit when the vegetation cover is not valued. To overcome this issue, the concept of phytomanagement combines risk mitigation through aided phytostabilisation, with a sustainable and profitable valorisation method for the produced biomass [1,12].

In that view, the cultivation of aromatic plants for the reclamation of TE-polluted lands appears as a promising approach. Intended for cultivation on marginal lands, they would not compete with feeding agriculture and as non-food crops, aromatic plants minimize the risk bound to the food-chain contamination [13]. In addition, the production of essential oils (EO), being high added-value biosourced products, could help producing an economic profit.

In the plant kingdom, the Lamiaceae family is one of the biggest flowering plants’ family, comprising a wide number of valuable aromatic species [14,15]. It has notably been demonstrated that some of them were displaying a tolerance to TE and hence could be valuable in phytomanagement approaches [13,16]. Clary sage (*Salvia sclarea* L.) is a biennial aromatic plant cultivated worldwide for its high-value EO, widely known for its use in perfumery and cosmetics but also for its applications in medicine [17,18]. Coriander (*Coriandrum sativum* L.) is another annual herbaceous aromatic plant, belonging to the Apiaceae [19,20]. It should be noted that EO composition could strongly be influenced, among other environmental factors, by soil characteristics, including the presence of pollutants [17,21]. It was in particular suggested that the presence of TE in soil resulted in modified EO yield, composition, and quality [22,23,24]. Likewise, the establishment of the mycorrhizal symbiosis has been reported as a variation factor for the EO composition and amount [25,26].

Historically used in traditional medicine, EO are nowadays arousing great interest, due to their various application fields [27,28,29]. EO could for instance be beneficial for human health and find applications as healthcare products, owing to their antioxidant and anti-inflammatory capacities [30,31]. In particular, one of the main concerns in healthcare problems resides in adverse effects of airborne particulate matter (PM) on human health.

Currently, synthetic antioxidants and anti-inflammatory compounds are suspected to be carcinogenic and responsible for health disorders [32,33]. Classified as “Generally Recognized As Safe” by the Food and Drug Administration, eco-friendly and well-accepted by consumers [32,34,35], EO are getting more and more attractive and could be particularly helpful as alternatives to synthetic agents.

Nevertheless, EO are very susceptible to degradation and possess low aqueous solubility and bioavailability, which could be challenging for biological applications. To overcome this issue, the use of a *β*-cyclodextrin (*β*-CD), a cyclic oligosaccharide allowing the encapsulation of hydrophobic compounds in aqueous solutions, is a promising avenue [33,36,37].

To our knowledge, most of the studies conducted on polluted soil until now consider either bioenergy production or renewable materials as valorisation channels for the produced biomass. In this study, the focus was put on an innovative valorisation channel based on the production of EO originating from aromatic plants’ cultivation, combined with an aided phytostabilization channel, relying on the use of a microbial inoculant consisting of an arbuscular mycorrhizal fungus species. This report particularly focuses on investigating EO valorisation, with a non-food perspective. The evaluation of EO’s biological properties related to fields such as healthcare products could help identify innovative valorisation channels, in view of evaluating and legitimating the channel method’s sustainability. Thus, the aim of this study was first to investigate the potential influence of the soil pollution by TE and of the mycorrhizal inoculation on the chemical composition of the EO distilled from clary sage and from both aerial parts and seeds of coriander and on their biological properties in the presence or in the absence of *β*-CD. Their anti-inflammatory and antioxidant properties were assessed, so as to investigate their potential use as healthcare products.

## 2. Results

### 2.1. Determination of the EO Chemical Composition

The analysis of EO chemical composition revealed that EO from seeds of coriander and sage contained mostly terpenes, whereas EO from the aerial parts of coriander contained mostly aldehydes and some terpene compounds. A total of 22 compounds were identified in sage EO, while 11 and 15 compounds were identified in the EO of coriander from aerial parts and seeds, respectively. Linalool was the only compound found in all distilled EO, and was particularly abundant in coriander seeds’ EO (ranging from 75 to 83%—Table 1). (Z)-2-decenal and linalool were the two compounds presenting the greatest proportions (ranging from 37.9 to 51.4% and from 21.8 to 39.2%, respectively) in coriander aerial parts’ EO, whereas linalyl acetate and germacrene D were the two main compounds in sage EO (ranging from 50.6 to 63% and from 6.7 to 17.1%, respectively).

**Figure 1:**
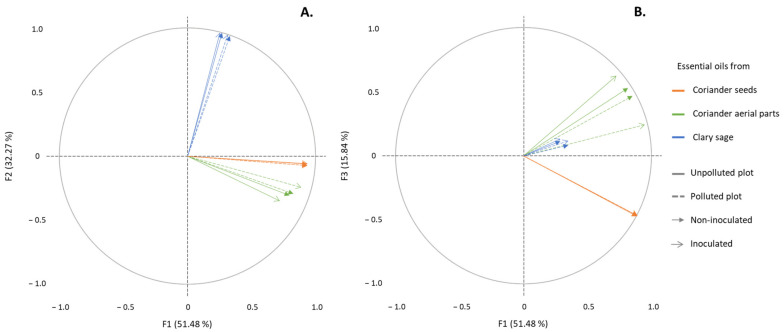
Correlation circles from the PCA analyses based on chemical composition of EO from aerial parts or seeds of coriander and from clary sage, with projection on F1 and F2 axes (**A**) and on F1 and F3 axes (**B**).

A PCA analysis was carried out and represented by means of correlation circles (Figure 1A,B). The first two principal components, F1 and F2, represented 83.7% of the initial variability of the data. In order to avoid misinterpretation, an additional projection on F1 and F3 (67.3% of the initial variability of the data) was executed. A strong correlation, depicted by vectors with highly similar directions and high correlation coefficients (data not shown), correspond to highly similar chemical compositions. Conversely, orthogonal vectors, and close to null correlation coefficient correspond to highly different chemical compositions.

The results show that EO from aerial parts of coriander, regardless of the condition (polluted or unpolluted sites, I or NI conditions), were strongly correlated together (Figure 1A,B—correlation coefficients between 0.914 and 0.995, *α* = 0.05—data not shown). Similarly, EO from seeds of coriander (correlation coefficients equal to 0.999, *α* = 0.05—data not shown) and from sage (correlation coefficients between 0.972 and 0.999, *α* = 0.05—data not shown) were strongly correlated. As such, no significant differences were observed between EO from a same plant part under the different experimental conditions (polluted or unpolluted sites, I or NI conditions). Besides, sage EO were not correlated to any EO from coriander, while EO from aerial parts and seeds of coriander were slightly correlated together (correlation coefficients ranging from 0.344 to 0.695, *α* = 0.05).

### 2.2. Determination of the Retention Capacity of EO by β-CD

All the EO evaluated in this study were able to be encapsulated in *β*-CD. EO retention percentages by *β*-CD are listed in Table 2.

The obtained EO retention percentages by *β*-CD were ranging from 63 to 81%. There were no significant differences in terms of EO retention between all the EO distilled from aerial parts of coriander, ranging from 72 to 75%, or from seeds of coriander, varying from 73 to 76%. However, significant differences were observed between sage’s EO, especially between the EO distilled from the biomass obtained on the NI unpolluted (63% retention) and polluted conditions (81% retention). Additionally, there is no difference between the I and NI conditions, except for clary sage EO originating from the polluted plot.

### 2.3. EO Antioxidant Activity

**Figure 2:**
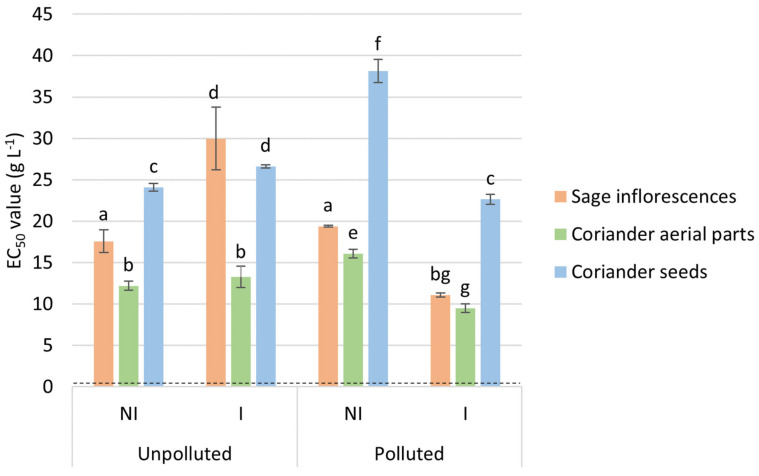
EO’s EC_50_ values (g L^−^^1^) resulting from the DPPH^•^ scavenging assay. The EC_50_ value obtained for Trolox^®^ (dotted line), was used as a positive control. Values are means ± SD (*n* = 3). Means followed by the same lowercase letter do not differ significantly by Two-way ANOVA test (*α* = 0.05).

Antioxidant activity of the different tested EO was evaluated through a DPPH^•^ scavenging assay, displaying EC_50_ values ranging from 9 to 38 g L^−1^ (Figure 2). These results indicated that, given the lower EC_50_ values that were obtained, coriander aerial parts’ EO showed the highest free radical scavenging ability, in comparison with sage and coriander seeds EO. Their efficiency was also compared to a well-known antioxidant standard, namely Trolox^®^, and expressed in terms of TEAC, inversely proportionate to the EC_50_ values obtained. EO from coriander aerial parts displayed TEAC higher than 1 µmol Trolox^®^.g^−1^ of EO, while, comparatively, those from coriander seeds and sage showed TEAC ranging from 0.4 to 0.9 µmol Trolox^®^ g^−1^ of EO (results not shown). The antioxidant capacity increases in the following order: coriander seeds EO < sage EO < coriander aerial parts EO < Trolox^®^. Additionally, significant differences were obtained between the different experimental conditions (NI or I, unpolluted or polluted site) for each evaluated EO (coriander seeds or aerial parts and sage). Namely, EO originating from the I condition on the polluted site displayed lower EC_50_ values in comparison with those from the NI condition (regardless of the EO), while the trend is the opposite on the polluted site, except for the EO from aerial parts of coriander. Besides, no clear trend could be highlighted between polluted and unpolluted sites.

### 2.4. EO Anti-Inflammatory Activity

#### 2.4.1. Cytotoxicity Assessment of EO

**Figure 3:**
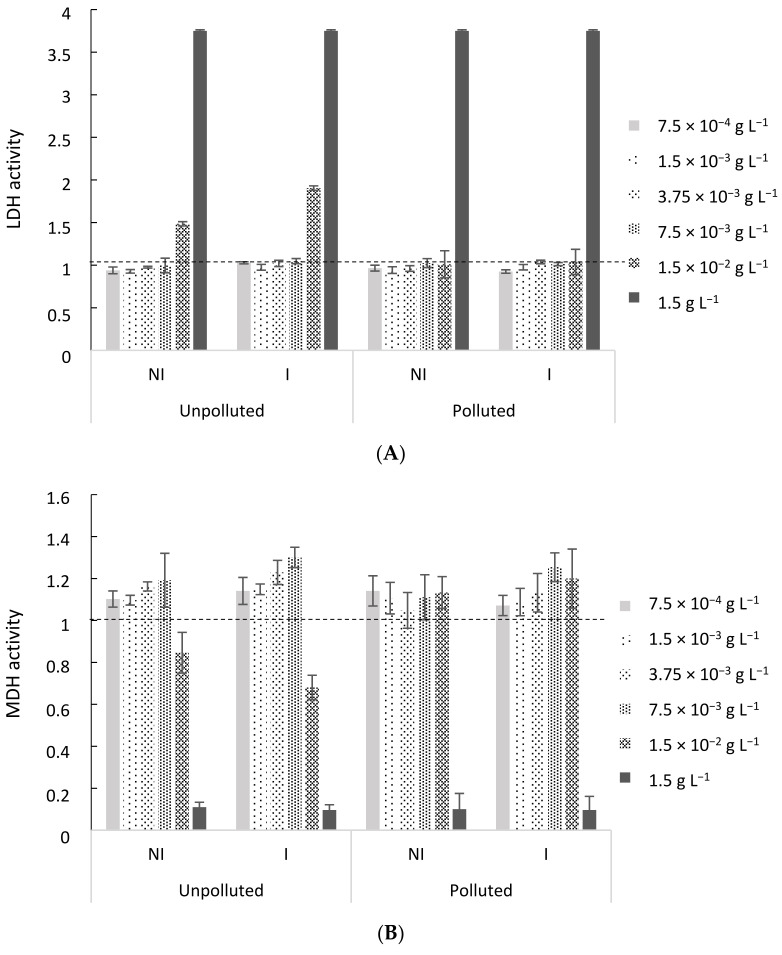
Lactate dehydrogenase (LDH—**A**) and mitochondrial dehydrogenase (MDH—**B**) activities in cell-free culture supernatants of BEAS-2B cells, after 48 h of exposure to coriander aerial parts EO at different concentrations. Cells exposed to the culture medium only were used as a negative control (dotted line). Results are depicted as mean ± SD of 3 replicates for each exposure condition.

The first step in the assessment of the potentially beneficial anti-inflammatory effects of the three tested EO is the determination of the concentration at which no cell death is observed. To do so, the cytotoxicity of EO from coriander aerial parts (Figure 3A,B), coriander seeds and sage (results not shown) was evaluated through LDH and MDH assays. Important cytotoxic effects were observed for both LDH and MDH assays at the highest tested concentration (1.5 g L^−1^ of EO), while the first cytotoxic effects appeared at a concentration of 1.5 × 10^−2^ g L^−1^ of EO for coriander aerial parts EO, regardless of the experimental conditions. At the lowest tested concentrations, up to 7.5 × 10^−3^ g L^−1^, no cytotoxic effect was observed. Similar results were obtained for EO from coriander seeds and sage. In that regard, two EO concentrations of 1.5 × 10^−3^ and 7.5 × 10^−3^ g L^−1^ were chosen for further evaluation of the EO anti-inflammatory effects, regardless of the EO, as they exhibited a similar response in terms of cytotoxicity.

#### 2.4.2. Anti-Inflammatory Properties of Free or Encapsulated EO

**Figure 4:**
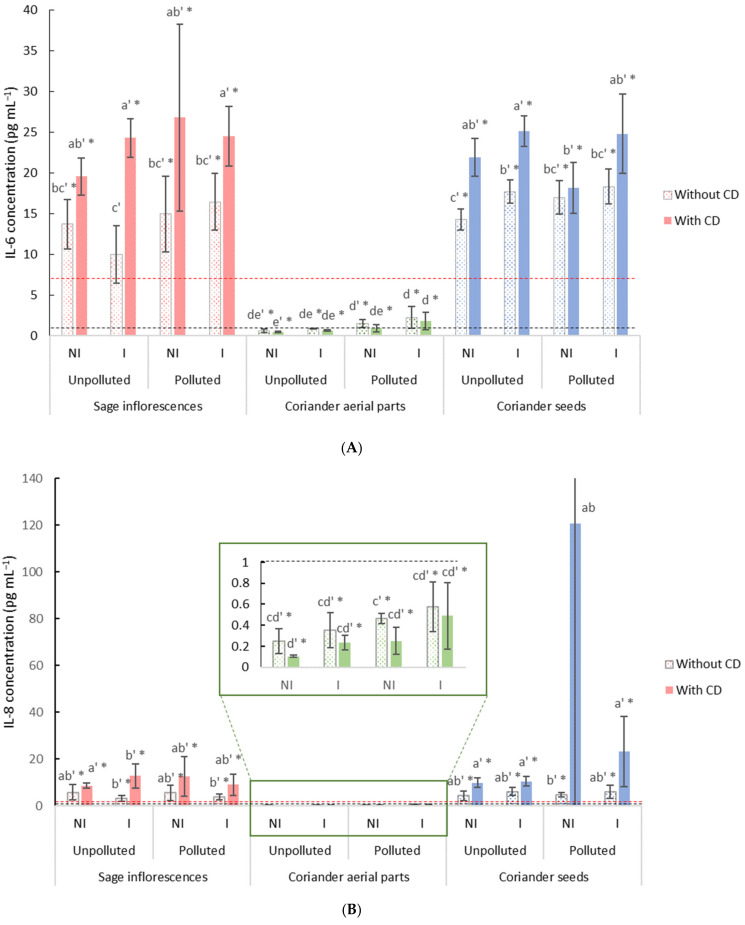
Cytokine levels in BEAS-2B cells exposed for 48 h to PM_2.5_ in the presence of free or *β*-CD encapsulated EO: (**A**) IL-6 concentrations in BEAS-2B cells, (**B**) IL-8 concentrations in BEAS-2B cells. The negative and positive control values are represented by the black and red dotted lines, respectively. Data are presented as mean ± standard deviation (*n* = 3). Significant differences using a two-way ANOVA comparison (*α* = 0.05), between results obtained in free *β*-CD condition and in the presence of *β*-CD, are indicated by different lowercase letters, differences between experimental conditions and the negative control are displayed with an apostrophe (’), while significant difference with the positive control are displayed with an asterisk (*-*p* < 0.05). CD: Cyclodextrins; BEAS-2B: human bronchial epithelial cells; IL-6: interleukin 6; IL-8: interleukin 8; PM_2.5_: particulate matter with an aerodynamic diameter inferior to 2.5 µm.

After 48 h of exposure to PM_2.5_ (15 µg cm^−^^2^), with or without the addition of free or *β*-CD-encapsulated EO, the concentrations of IL-6 (Figure 4A) and IL-8 (Figure 4B) were measured and compared to their respective controls. The results obtained for the EO of coriander aerial parts exhibited a reduction in the inflammation, as depicted by the significant decrease observed in IL-8 levels in comparison with the positive control and that regardless of the condition (Figure 4B). Conversely, in the case of EO from sage and coriander seeds, IL-6 and IL-8 levels were significantly higher, up to four times, in comparison with the positive control.

In the case of coriander aerial parts EO, in the presence of *β*-CD, levels of both IL-6 and IL-8 were not modified, indicating that EO encapsulation did not have any effect on the EO’s anti-inflammatory effect. However, regarding the EO of sage and coriander seeds in the presence of *β*-CD, higher levels of IL-6 in particular were measured in most of the experimental conditions in comparison with both the positive control and the *β*-CD free condition. Besides, regarding both IL-6 and IL-8 levels, no clear trend in terms of anti-inflammatory capacity was observed for EO distilled from a same plant part but under the different experimental conditions.

## 3. Discussion

This study was carried out in the framework of a phytomanagement approach, aiming at cultivating high economic value aromatic plants on TE-polluted soils, to initiate the reclaiming of such areas [41]. In this work, the influence of the soil pollution by TE and mycorrhizal inoculation on the EO chemical compositions were first assessed. The potential of EO distilled from clary sage (inflorescences) and coriander (aerial parts and seeds) regarding their antioxidant and anti-inflammatory activities were also investigated *in vitro*, in the presence or in the absence of *β*-CD.

### 3.1. Effects of Soil Pollution by TE and Mycorrhizal Inoculation on Chemical Compositions of EO Distilled from Clary Sage Inflorescences and Coriander Aerial Parts and Seeds

Although the relative abundances may slightly vary, all previous reports on clary sage EO seem to agree on the EO composition, highlighting the monoterpenes linalyl acetate and linalool as major compounds [17,21,42]. These were the two compounds that were obtained at the highest in these experimental conditions. Clary sage EO is indeed mostly constituted of oxygenated monoterpenes, up to 85% of the EO composition, followed by sesquiterpenes. Besides, relatively high proportion of germacrene-D (up to 17%) were obtained in this work. This chemotype of clary sage EO matches with one that was previously reported [17,43]. Similarly, data regarding the composition of EO from coriander aerial parts and coriander seeds are in line with previous reports, depicting the prevalence of alcohols and aldehydes in the EO from aerial parts [19,20,38,44], while the EO from coriander seeds was mostly constituted of monoterpenes [19,44]. In fact, these results demonstrated a high proportion of linalool (up to 83%) in the EO from coriander seeds and lesser fractions of *α*-pinene (from 3 to 5%) and *γ*-terpinene (from 6 to 9%). As previously reported [19,44], the EO from coriander aerial parts was significantly different from the one distilled from the seeds, largely composed of 2-decenal (up to 50%), linalool, decanal and 2-dodecenal. A similar trend has also been demonstrated for several other plant species, mainly from the Apiaceae, including *Foeniculum vulgare* Mill., *Laser tribolium* L. or *Prangos uloptera* DC [45,46,47], but also from the Lamiaceae, such as *Stachys schtschegleevi* Sosn. [48].

In this study, the soil pollution by TE did not significantly alter EO composition. This result is not in agreement with some previous studies, which showed that the presence of TE in soil, among other environmental conditions, was susceptible to affecting EO composition and quality [22,24]. Notably, it was suggested that the stress induced by the presence of TE resulted in a modification of the secondary metabolism [24,49,50,51]. This could either lead to an inactivation of enzymes involved in the EO biosynthesis pathways, in the case of *Mentha arvensis* L., *Mentha piperita* L. and *Vetiveria zizanioides* Linn. Nash [23,24,49], or to an elicitation of specific biosynthesis pathways (*Mentha spicata* L., *Mentha pulegium* L. or *Cymbopogon flexuosus* Nees) in response to TE exposure [16,50,51]. It should be noted that the response greatly varies from one aromatic plant species to another and depends on the TE involved [22,24]. Specifically, Cd, Cr, Ni and Pb exposure, up to 75 ppm of each TE, were tested [24]. The application of Cr, Ni and Pb displayed deleterious effects on both EO yield and composition. Whereas Cr and Ni amounts are significantly greater than those in this study, and hence could explain the difference in terms of plant response to TE exposure, Pb concentration was significantly lower, and led to similar effects [24]. Several other studies that have been conducted under TE stress and synthesized in a previous review [22], concluded that increasing concentrations of TE (Cd, Cr, Pb or Cu), within the same range as those tested, led to a decrease in EO yield and affected EO composition. Besides, aromatic plants from the Lamiaceae family and the genus *Salvia* in particular were depicted as consistently growing even in TE-polluted conditions, and displaying unaffected EO composition [22,52], corroborating the obtained results.

In this work, mycorrhizal inoculation did not result in significant changes in the EO composition either, even though the colonization of plant roots by AMF has previously been reported as significantly modifying both the EO quantity and quality [25,26]. In the presence of the mycorrhizal symbiosis, the metabolism of the host plant has in fact been found modified [53,54,55]. However, it rather pointed towards an augmentation of the EO content, by affecting the qualitative and quantitative patterns of secondary metabolites synthesis [56]. It should be noted that in field experiments, inoculation using a commercial inoculum might result in very limited root colonization by AMF species [57]. The mycorrhizal colonization was evaluated in previous related studies, demonstrating that the mycorrhizal symbiosis was successfully established, even though it did not result in an improved plant growth [41,58]. In that respect, it could have led to non-significant changes in terms of EO composition.

### 3.2. EO Potential Use as Natural Therapeutic Products and Insights on the Relationships between EO Composition and Their Biological Effects

Data regarding antioxidant activity showed that all the tested EO revealed a clear antioxidant potential, but displayed different ranges of efficiency, with IC_50_ ranging from 9 to 38 g L^−1^. These indicated that the EO from aerial parts of coriander possessed the highest free radical scavenging capacity using the DPPH^•^ scavenging assay. These results are in line with results found in the literature, depicting IC_50_ values ranging from 0.001 to 1.5 g L^−1^, depending on the tested EO, including *Allium cepa* L., *Citrus aurantium*, *Myrtus communis,* and *Eucalyptus oleosa* EO [59,60,61,62]. So as to compare the antioxidant capacity of the EO to that of a well-known antioxidant standard, Trolox^®^, the obtained results were also expressed in terms of TEAC. TEAC values ranged from 0.4 up to 1.62 µmol Trolox^®^ g^−1^ of EO, for coriander aerial parts and seeds EO, respectively. This indicated that EO from coriander aerial parts exerted an antioxidant capacity slightly higher than the two other EO, while sage and coriander seeds EO showed a limited antioxidant capacity. This result is in agreement with previous findings on coriander aerial parts EO, which showed an antioxidant potential of the EO demonstrated by an IC_50_ value lower than that of the reference synthetic antioxidant compound [34]. These findings suggest that the EO from coriander aerial parts in particular could be of interest to prevent lipid oxidation and reactive oxygen species (ROS) generation into the human body, preventing oxidation damage [34,63]. It has been hinted that natural antioxidant, such as EO, can avoid ROS formation by acting as physical barriers, preventing the access to biological sites and binding metallic ions [63]. Moreover, they were also shown capable of scavenging and inactivating ROS [63].

In addition to their ability to scavenge free radicals and prevent ROS formation, the EO were also tested for their anti-inflammatory properties. As previously shown, BEAS-2B cells exposed to PM_2.5_ exhibited an inflammatory response, when compared to control cells [64,65]. The response of the BEAS-2B cells to the addition of the EO varied, since coriander aerial parts’ EO drastically decreased the levels of secreted IL-8, exhibiting a strong anti-inflammatory effect. Conversely, the addition of clary sage and coriander seeds’ EO led to the opposite effect, with concentrations of interleukins up to four times higher than the control. Hence, only the EO from aerial parts of coriander exerted an anti-inflammatory effect, protecting the cells against the stress induced by PM_2.5_ intoxication.

Altogether, the obtained results suggest that the presence of TE in soil did not alter the EO biological effects, regardless of the assessed property. Evaluated for antioxidant and anti-inflammatory applications, EO originating from the biomass cultivated on the polluted site displayed similar efficiencies to the one distilled from the unpolluted one. This finding goes along with the results obtained in terms of chemical compositions, which were found similar between unpolluted and polluted conditions. Likewise, the mycorrhizal inoculation did not lead to significant changes in the EO’s effects, regardless of the biological property. This result is also consistent regarding the obtained EO compositions, which were similar between inoculated and non-inoculated conditions in this study.

It was previously suggested that mono- and sesquiterpenes present in the EO were responsible for the EO antimicrobial, but also herbicidal, antioxidant and anti-inflammatory activities [20,34,38,66]. As an example, linalool is often brought forward as the main explanatory factor of the EO activity [34]. However, in this work, linalool is present in all the tested EO in various proportions, which alone are not sufficient to explain the different ranges of efficiency that were assessed regarding the biological activities. These results suggest that it is important to consider the presence of minor compounds as well. It has in fact repeatedly been demonstrated that EO’s activities were the result of a synergism between the EO constituents, since the evaluation of the latter isolated often led to decreased performances [20,67]. In that regard, studying the mechanisms of action of the EO and of some of their compounds, alone or in combination, could help towards the understanding of the interconnection between the EO composition and the related biological activity.

On another note, EO bioavailability is often brought forward as limited. To overcome this issue, encapsulation in *β*-CD could favour EO solubilisation and allow a controlled-release of the EO [33,35]. *β*-CD encapsulation may hence be of great interest to address EO downsides [68,69,70]. In this study, all the tested EO showed a relatively high capacity to be efficiently complexed with *β*-CD, with retention percentages ranging from 63 to 80%. *β*-CD among other CD was in fact previously demonstrated to possess a higher retention capacity [33,71]. Interestingly, the measured retention capacities, regardless of the considered EO, were in the same range as what was previously described for *β*-CD but with other EO, such as *Citrus reticulata*, *Cymbopogon nardus*, *Origanum majorana*, *Rosmarinus officinalis* or *Satureja montana* [33]. These results suggest that *β*-CD could efficiently form inclusion complexes with the EO’s components and thus enhance their solubility.

However, in these experimental conditions, EO encapsulation in *β*-CD did not provide a significant enhancement in terms of EO efficiency for any of the tested biological activity, although it was previously demonstrated [72]. The encapsulation of EO could nonetheless provide an efficient tool to enhance the EO solubilization and diffusion throughout the organism and facilitate its accession to the target [65], and should be further explored *in vivo* to legitimate a potential use for healthcare applications.

## 4. Materials and Methods

### 4.1. Essential Oils

The EO oils tested in this study were obtained through the steam distillation of two aromatic species, namely coriander (*Coriandrum sativum* L.) and clary sage (*Salvia sclarea* L.), cultivated in situ on two experimental plots: a TE-polluted (50°25′55.5″ N, 3°02′25.5″ E) one, displaying elevated amounts of TE (Pb: 394 ppm—Zn: 443 ppm—Cd: 7.2 ppm) and an unpolluted one (50°49′55.7″ N, 1°55′46.9″ E). Experimental plots were inoculated (I) or not (NI) with a commercial inoculant containing the AMF *Rhizophagus irregularis* (AGTIV^®^ Specialty Crops, 125 g ha^−1^ Premier Tech, QC, Canada), kindly provided by Premier Tech. Full descriptions of the experimental sites and design, and plant physiological data, are provided in [41,58]. The distillation of the harvested plant biomass was performed during a three-hour cycle until no more EO was recovered on a steam-distillation unit (14 m^3^ tank-saturated water steam, 0.3 bar). Coriander’s distillation was performed using either the harvested aerial parts (full flowering stage) or the harvested seeds at maturity, whereas sage’s distillation was performed using harvested inflorescences at full blossoming during the second year of cultivation. EO were stored at 4 °C in tightly closed brown glass vials until their analysis.

### 4.2. Determination of the EO Chemical Composition

EO were analysed by electron ionization gas chromatography–mass spectrometry (QP 2010 Ultra, Shimadzu, Marne-la-Vallée, France). The system was operated using helium as a carrier gas at a constant linear velocity (60 cm s^−^^1^), as previously described [73]. Essential oil’s samples were first diluted in ethyl acetate (ratio 1:200 (*v*/*v*)) and 0.2 µL of the resulting solution was injected in a split mode (split ratio 1:10) in a ZB-5MS (5%-phenyl-arylene/95% dimethylpolysiloxane—10 m length, 0.10 mm inner diameter, 0.10 μm phase thickness—Phenomenex, Le Pecq, France) capillary column. The column temperature was programmed to linearly increase at a constant rate of 40 °C min^−1^, from 60 °C to 280 °C, held for 2 and 1 min, respectively.

Mass spectra were recorded with an ionisation energy of 70 eV and an interface temperature of 280 °C, on a mass range of 35.0 to 350 (*m*/*z*). Kovats indices were calculated from the retention times after co-injection with n-alkanes and the identification of individual EO compounds was carried out based on the comparison of their obtained mass spectra and kovats indices with those listed in the NIST (National Institute of Standards and Technology, Gaithersburg, MD, USA) and Wiley 275 computer libraries, as well as those found in the literature [17,19,21,38,42,74]. Relative percentages of oil constituents were measured from the GC peak areas.

### 4.3. Determination of the Retention Capacity of EO by β-CD

The capacity of the *β*-CD to encapsulate EO (retention capacity) was quantified through a static headspace-gas chromatography (SH-GC) analysis, performed according to [73]. Briefly, EO were added to 10 mL of either water or 10 mM aqueous *β*-CD solutions in 22 mL headspace glass vials. Suspensions were prepared by mixing β-CD, at a concentration of 10 mM, and EOs, in water. The solutions were then agitated until a homogeneous mixture was obtained, as previously described [70]. The vials were equilibrated at 25 ± 0.1 °C before analysis. Then, 1 mL of vapor present in the upper part of the vial at equilibrium was withdrawn by using a gas-tight syringe and injected directly into the chromatographic column. A total runtime of 32 min was required per sample (initial temperature of 50 °C for 2 min, increased to 190 °C at a constant rate of 5 °C min^−1^). All analyses and measurements were carried out using an headspace autosampler (Agilent, Les Ulis, France) and an Autosystem XL (Perkin Elmer, Courtaboeuf, France), equipped with an FID detector. A DB624 (6% cyanopropylphenyl/94% Dimethyl-polysiloxane, bonded and crosslinked phase—30 m length, 0.53 mm inner diameter and 3 µm phase thickness—Phenomenex, Le Pecq, France) chromatographic column was also used. The measurement of the retention capacity was expressed as follows:

(1) $$r\;(\%)\;=\left(1-\frac{\sum A_{\mathrm{CD}}\;}{\sum A_{0}}\right)\times 100$$

where ∑A_0_ and ∑A_CD_ stand for the sum of each EO component’s peak area in the absence and the presence of CD, respectively. Measurements were led in triplicates for each EO, in the presence or absence of the *β*-CD.

### 4.4. Biological Properties of EO

#### 4.4.1. Antioxidant Activity

The 2,2-diphenyl-1-picrylhydrazyl radical (DPPH*) scavenging assay was used to determine the antioxidant activity of the different EO, according to [75], followed with some minor modifications. An EO concentration scale (up to 33 g L^−1^) was prepared in ethanol for each and all of the tested EO. An amount of 1 mL of each ethanolic EO solution was then mixed with 2 mL of ethanolic DPPH* solution (0.1 mM). All mixtures were shaken for 1 h in the dark at 20 ± 1 °C. The absorbance was measured using a UV/Vis spectrometer (Lambda 2S, Perkin Elmer, Courtaboeuf, France) at 517 nm. The radical scavenging activity was evaluated using the following equation:

(2) $$\mathrm{Antioxidant}\;\mathrm{Activity}\;(\mathrm{AA})\;=\;\left(1-\;\frac{A_{S}}{A_{0}}\right)\times 100$$

where A_S_ and A_0_ stand for the absorbance of the sample and of the blank, respectively.

Obtained results for each EO were expressed as EC_50_ values, defined as the concentration of antioxidant that causes a 50% decrease in the DPPH^*^ absorbance, by graphical interpolation. Additionally, EO EC_50_ values were compared to those obtained for 6-hydroxy-2,5,7,8-tetramethylchroman-2-carboxylic acid (Trolox^®^, Sigma-Aldrich, Saint-Quentin-Fallavier, France), a water-soluble analog of vitamin E, with results expressed as TEAC (Trolox^®^ equivalent antioxidant capacity).

#### 4.4.2. Anti-Inflammatory Activity

##### Cell Lines and Culture Conditions

Human bronchial epithelial cells (BEAS-2B) were cultured in CellBIND^®^ surface plastic flasks (Corning—Thermo Fisher Scientific, Illkirch, France) in LHC-9 medium and incubated at 37 °C in a humidified atmosphere containing 5% of CO_2_ [65].

##### EO Cytotoxicity Assessment

The EO cytotoxicity was first assessed at different EO concentrations (i.e., 7.5 × 10^−4^ to 1.5 g L^−1^) using the BEAS-2B cell line. BEAS-2B cells, exposed or not to the different EO, were seeded in 96-well CellBIND^®^ microplates at a density of 20,000 or 10,000 cells/200 µL medium for 24 or 48 h of exposure, respectively. Cytotoxicity was then evaluated by measuring the extracellular lactate dehydrogenase activity in supernatants (Cytotoxicity Detection Kit LDH, Roche Diagnostics, Neuilly-sur-Seine, France), and mitochondrial dehydrogenase (MDH) activity (Cell Proliferation Reagent WST-1, Roche Diagnostics, France), as previously described [64,65]. Cells not exposed to EO and exposed to Triton X-100 (2%) were used as negative and positive controls, respectively.

##### Cell Exposure for the Study of EO Anti-Inflammatory Potential

The anti-inflammatory potential of EO was assessed through a method adapted from [35]. BEAS-2B cells were previously seeded in 96-well CellBIND^®^ microplates, in order to obtain 40,000 cells/well at the end of the exposure time. Cells were then exposed to PM_2.5_, i.e., particulate matter with an aerodynamic diameter inferior to 2.5 µm (15 µg cm^−^^2^) to induce an inflammatory response [64,76]. Particles were prepared in the LHC-9 culture medium at the desired concentration in order to reach the final concentration (i.e., 15 µg cm^−2^) in the well, and sonicated for 5 min before intoxication for a better homogenization [76,77]. The microplates were then incubated for 48 h, in the presence of EO at the two tested concentrations (1.5 × 10^−3^ and 7.5 × 10^−3^ g L^−1^), either free or encapsulated in *β*-CD (0.1 mM). Cells unexposed to EO and unexposed to both EO and PM_2.5_ were used as positive and negative controls, respectively. After 48 h of exposure, aliquots of cell-free culture supernatants were collected and frozen (−80 °C) for the determination of cytokines’ concentrations and total protein content analysis. Cytokines’ concentrations were determined by a sandwich-based Elisa analysis (Quantikine ELISA Kits, R&D Systems Europe, Ltd., Abingdon, UK) and the total protein contents in cell-free culture supernatants were quantified using the BiCinchoninic Acid kit (Sigma-Aldrich, Saint-Quentin-Fallavier, France). Two interleukins, namely IL-6 and IL-8, were selected as cytokines of interest for the assessment of the anti-inflammatory potential of the tested EO, given their known implication as pro-inflammatory mediators in cells exposed to atmospheric particles [34,65].

### 4.5. Statistical Analyses

Statistical analyses were performed using XLSTAT 2018.1.1 (Adinsoft, Paris, France) software and R 3.6.1 [78]. Prior any statistical analysis, Shapiro-Wilk and Bartlett tests were performed on the data to verify the normality and the homoscedasticity assumptions, respectively. When necessary, non-normal data were “square-root” or “log10” transformed.

For both biological properties evaluated, IC_50_ or EC_50_ values resulted from non-linear regression analyses from triplicate assays and were expressed as mean values and standard deviation (mean ± SD). The comparison of IC_50_ or EC_50_ values was carried out using two-way analysis of variance (ANOVA), complemented with a post-hoc Tukey-HSD (Honestly Significant Difference) test. Interleukins’ concentrations resulting from the different experimental conditions were also subjected to two-way ANOVA and Tukey-HSD tests.

Moreover, a Principal Component statistical Analysis (PCA) was carried out to identify the possibly existing correlations between the different EO and to investigate the separation between EO samples according to their main chemical constituents.

## 5. Conclusions

Altogether, the above findings have shown that the three tested EO, from clary sage (inflorescences) and coriander (aerial parts and seeds), displayed faithful chemical compositions, despite the soil pollution by TE and the addition of a mycorrhizal inoculant.

They were also demonstrated to possess valuable antioxidant and anti-inflammatory activities. Notably, the results showed that the EO distilled from aerial parts of coriander possess a higher efficacy, regardless of the tested biological activity. Afterwards, if such *in vitro* assays allowed a first screening of the EO biological activities and may indicate their potential towards applications as natural therapeutic products, further *in vivo* assays should be carried out to validate EO-based products’ efficacy. In addition, these EO could be tested alone or in association, to investigate potential synergistic effects, as well as in combination with conventional marketed products, so as to reduce the amounts used. Besides, in these experimental conditions, even though the encapsulation of the tested EO in *β*-CD did not significantly improve the EO efficiency, *β*-CD efficiently formed inclusion complexes with the EO, which could enhance their solubilisation as well as their bioavailability. Further assessments should be conducted to confirm the capacity of *β*-CD to act as a green solubilizer while preserving the encapsulated compounds’ activity in *in vivo* conditions.

From a wider perspective, this study supports the idea that aromatic plant cultivation on TE-polluted areas and EO distillation from the produced biomass can be a means to reclaim these marginal sites. In particular, EO are high added value products and as depicted in this work and could be valued for their biological properties, provided that the bioavailability and target accession issues are addressed. In addition, their market globally and steadily increases, offering economic opportunities.

## Figures and Tables

**Table 1:** Chemical composition of the EO distilled from aerial parts or seeds of coriander and from sage inflorescences. Data are relative percentages of EO compounds.

Retention Indexes		Distilled Plant	Aerial Parts of Coriander				Seeds of Coriander				Sage Inflorescences			
		Biomass Origin	Unpolluted		Polluted		Unpolluted		Polluted		Unpolluted		Polluted	
Exp.	Lit.	EO Compounds	NI	I	NI	I	NI	I	NI	I	NI	I	NI	I
908	909	***α*-pinene**	0.9	1.3	1.0	1.3	5.4	4.5	3.3	2.7	-	-	-	-
944	945	**Camphene**	-	-	-	-	0.6	0.5	0.4	0.3	-	-	-	-
991	991	***β*-myrcene**	-	-	-	-	-	-	-	-	2.1	1.5	0.9	1.2
1005	1004	**4-carene**	-	-	-	-	0.3	-	-	0.8	0.2	0.1	0.1	0.2
1027	1031	**Limonene**	-	-	-	-	1.7	1.6	1.3	1.7	0.8	-	0.5	0.1
1034	1034	**p-cymene**	0.3	0.3	0.2	1.1	1.6	1.6	1.3	1.2	-	-	-	-
1040	1036	***β*-phellandrene**	0.7	-	-	-	-	-	-	-	0.2	0.1	0.1	0.1
1049	1051	**Ocimene**	-	-	-	-	-	-	-	-	0.7	0.6	0.4	0.7
1065	1066	**γ-terpinene**	1.6	1.9	1.4	1.8	8.9	8.6	6.6	8.4	-	-	-	-
1100	1103	**Linalool**	29.3	21.8	32.1	39.2	75.0	76.8	83.2	79.3	10.0	10.4	16.5	14.8
1133	1140	**Camphor**	1.3	1.0	1.4	1.7	3.7	3.5	3.6	3.7	-	-	-	-
1193	1200	***α*-terpineol**	-	-	-	-	-	-	-	-	1.9	1.4	1.8	2.4
1205	1207	**Decanal**	6.6	7.9	7.2	8.0	-	-	-	-	-	-	-	-
1250	1253	**Linalyl acetate**	-	-	-	-	-	-	-	-	53.0	50.6	63.0	62.6
1274	1278	**(Z)-2-decenal**	48.0	51.4	47.1	37.9	-	-	-	-	-	-	-	-
1308	1310	**Undecanal**	-	-	3.5	0.9	-	-	-	-	-	-	-	-
1371	1371	**2-undecenal**	1.2	1.7	-	1.2	-	-	-	-	-	-	-	-
1375	1377	***α*-copaene**	-	-	-	-	-	-	-	-	3.6	4.5	1.9	2.0
1383	1383	**Geranyl acetate (cis)**	-	-	-	-	-	-	-	-	1.3	0.9	0.7	1.0
1386	1386	**Geranyl acetate (trans)**	-	-	-	-	1.6	3.0	0.3	1.8	2.5	2.1	1.5	2.5
1389	1390	***β*-cubebene**	-	-	-	-	-	-	-	-	0.2	-	0.1	0.1
1414	1418	***β*-caryophyllene**	-	-	-	-	-	-	-	-	3.2	3.9	2.6	2.0
1420	1420	**Dodecanal**	0.5	0.6	0.8	0.8	-	-	-	-	-	-	-	-
1427	1430	***β*-copaene**	-	-	-	-	-	-	-	-	1.1	1.4	0.7	0.5
1447	1450	***β*-farnesene**	1.5	1.4	0.3	0.0	0.8	-	-	-	-	0.1	0.1	-
1467	1468	**2-dodecenal**	4.6	5.8	3.0	3.7	-	-	-	-	-	-	-	-
1479	1480	**Germacrene D**	-	-	-	-	-	-	-	-	14.8	17.1	6.7	7.3
1484	1484	***α*-Humulene**	-	-	-	-	-	-	-	-	0.4	0.4	0.1	0.2
1515	1518	**Tridecanal**	-	0.9	-	-	-	-	-	-	-	-	-	-
1523	1523	***β*-cadinene**	-	-	-	-	-	-	-	-	1.2	1.6	0.4	0.6
1551	1556	**Germacrene B**	-	-	-	-	-	-	-	-	1.7	2.0	0.9	0.9
1570	1571	**2-tridecenal**	3.6	4.0	2.0	2.4	-	-	-	-	-	-	-	-
1580	1581	**Caryophyllene oxide**	-	-	-	-	0.2	-	-	-	0.3	0.4	0.2	0.2
1900	1900	**Sclareol oxide**	-	-	-	-	-	-	-	-	0.4	0.6	0.3	0.4
2220	2220	**Sclareol**	-	-	-	-	-	-	-	-	0.3	0.3	0.4	0.3
**Monoterpene hydrocarbons**			3.2	3.2	2.4	3.1	16.8	15.2	11.6	14.0	3.9	2.3	2.0	2.2
**Oxygenated monoterpenes**			30.6	22.8	33.5	40.9	80.5	83.2	87.1	84.8	69.0	65.3	83.5	83.2
**Oxygenated diterpene**			-	-	-	-	-	-	-	-	0.3	0.3	0.4	0.3
**Sesquiterpene hydrocarbons**			1.5	1.4	0.3	-	0.8	-	-	-	26.2	31.0	13.6	13.7
**Oxygenated sequiterpenes**			-	-	-	-	-	-	-	-	-	-	-	-
**Aromatic hydrocarbons**			0.3	0.3	0.2	1.1	1.6	1.6	1.3	1.2	-	-	-	-
**Aliphatic aldehydes**			64.4	72.3	63.5	54.9	-	-	-	-	-	-	-	-
**Terpenic oxide**			-	-	-	-	0.2	-	-	-	0.6	1.0	0.5	0.6

Exp.: experimental retention time; Lit.: retention time from the literature [21,38,39,40]; “-“: undetected compound; NI: Non-inoculated condition; I: Inoculated condition.

**Table 2:** EO’s percentages of retention in the presence of *β*-CD. Values are expressed as means ± SD (*n* = 3). For each plant part, means followed by an asterisk “*” are significantly different, by one-way ANOVA test (*α* = 0.05).

Biomass Origin	Distilled Plant Part	Aerial Parts of Coriander	Seeds of Coriander	Sage Inflorescences
Unpolluted plot	NI	74 ± 1.4	73 ± 1.0	63 ± 7.1
	I	72 ± 1.4	74 ± 2.1	63 ± 5.7
Polluted plot	NI	75 ± 2.8	76 ± 2.1	81 ± 3.5 *
	I	74 ± 1.4	73 ± 1.0	63 ± 7.1

NI: Non-inoculated condition; I: Inoculated condition.

## Data Availability

The original contributions presented in the study are publicly available.

## Sample Availability

Samples of the compounds are not available from the authors.
